# Psychiatric disorders and comorbidity in a Spanish sample of prisoners at the end of their sentence: Prevalence rates and associations with criminal history

**DOI:** 10.3389/fpsyg.2022.1039099

**Published:** 2023-01-12

**Authors:** Mireia Pagerols, Sergi Valero, Lourdes Dueñas, Rosa Bosch, Miquel Casas

**Affiliations:** ^1^Programa MIND Escoles, Hospital Sant Joan de Déu, Institut de Recerca Sant Joan de Déu, Esplugues de Llobregat, Spain; ^2^Unitat de Farmacologia, Facultat de Medicina i Ciències de la Salut, Departament de Fonaments Clínics, Universitat de Barcelona (UB), Barcelona, Spain; ^3^Ace Alzheimer Center Barcelona, Universitat Internacional de Catalunya, Barcelona, Spain; ^4^Networking Research Center on Neurodegenerative Diseases (CIBERNED), Instituto de Salud Carlos III, Madrid, Spain; ^5^Programa Reincorpora “la Caixa”, Departament de Justícia, Centre d’Iniciatives per a la Reinserció (CIRE), Generalitat de Catalunya, Barcelona, Spain; ^6^CIBER de Salud Mental (CIBERSAM), Instituto de Salud Carlos III, Madrid, Spain; ^7^Departament de Psiquiatria i Medicina Legal, Universitat Autònoma de Barcelona (UAB), Bellaterra, Spain

**Keywords:** substance use disorders (SUD), attention deficit/hyperactivity disorder (ADHD), learning disorders (LD), repeat incarceration, violent offending, community reintegration

## Abstract

**Introduction:**

This study examined, for the first time, the prevalence of mental disorders and comorbidities among inmates who were about to be released, and their association with criminal history.

**Methods:**

A Spanish sample of 140 prisoners at the end of their sentence was recruited from an occupational program. Psychiatric disorders were determined according to the Diagnostic and Statistical Manual of Mental Disorders criteria. Bivariate analyses followed by multivariate regression models were conducted to identify significant variables for repeat incarceration and violent offending.

**Results:**

The lifetime prevalence of Axis I disorders was 81.4%, with substance use disorders (SUD) and attention deficit/hyperactivity disorder (ADHD) being the most common diagnoses (51.4 and 31.4%, respectively). The current prevalence of Axis I disorders was 59.0%, including learning disorders (38.6%), ADHD (16.4%), and SUD (5.71%) among the most frequent syndromes. Thirty-six (26.5%) participants met criteria for a current Axis II disorder, which commonly was an antisocial personality disorder (12.5%). The majority of the sample (60.8%) suffered from two or more comorbid disorders during their lifetime, although the current prevalence fell to 23.3%. Childhood ADHD increased the number of imprisonments, while inmates convicted of a violent crime were more likely to present a learning disorder. Having a lifetime diagnosis of SUD or multiple psychiatric disorders appeared to be associated with both repeat incarceration and violent offending.

**Conclusion:**

Given the high rate of mental disorders still present among subjects completing prison sentences and the challenges they may encounter to benefit from vocational programs, our results suggest that appropriate psychiatric care should be provided during imprisonment and after release to facilitate their community reintegration.

## Introduction

According to the Council of Europe Annual Penal Statistics (SPACE I), on 31st January 2020 there were 1,528,343 inmates in the penal institutions of the Council of Europe member states (Aebi and Tiago, 2021). About 95% of them will eventually be released, although research suggests that nearly half return to correctional systems within the first year because they lack the skills to successfully transition from prison to community (Hughes and Wilson, 2002; Petersilia, 2005; Eggers et al., 2006). Employment has proven to be one of the most effective means for promoting the reintegration of offenders as it provides individuals with a legitimate source of income, a structured routine, certain status, and the opportunity to associate with law-abiding peers (Bahr et al., 2010; Peled-Laskov et al., 2019). Therefore, rehabilitation programs focused on professional training and developing the necessary skills for workplace settings are highly popular in the Western world and have been associated with multiple benefits, including higher self-esteem, better jobs opportunities upon release, and lower recidivism rates (Peled-Laskov et al., 2019; Zhao et al., 2019).

However, psychiatric disorders, which frequently entail emotional disturbances and cognitive limitations, such as attention deficits and learning difficulties, can significantly jeopardize a prisoner’s ability to successfully engage in interventions intended to increase employability (Lee et al., 2018). Of note, epidemiological studies have consistently demonstrated that mental illnesses are highly overrepresented among incarcerated adults (Fazel and Danesh, 2002; Fazel and Seewald, 2012). Indeed, a previous meta-analysis, based on pooled data from 33,588 prisoners in 24 countries, estimated that 65.0% of the male inmates were diagnosed with a personality disorder, which commonly was an antisocial personality disorder, 10.2% had major depression, and 3.6% experienced psychotic disorders (Fazel and Seewald, 2012). Attention deficit/hyperactivity disorder (ADHD) and substance use disorders (SUD) also appear to be overrepresented in adult prison populations, with prevalence rates around 25.0 and 50.0%, respectively (Mumola and Karberg, 2006; Young et al., 2015). Additionally, mental health problems are often found to coexist and subjects with multiple conditions feature more severe symptoms, greater functional impairment, less social competence, higher public service utilization, and worse treatment outcome (van Buitenen et al., 2020). Likewise, incarcerated offenders who present co-occurring disorders usually show more serious offending behaviors, are at higher risk of recidivism or repetition of criminal activity after release, and thus may struggle to achieve successful community reintegration (Chang et al., 2015; White et al., 2016; Kim et al., 2017; Garofalo et al., 2018; van Buitenen et al., 2020). In this sense, Fazel and Seewald (2012) reported that up to 43.5% of prisoners with any mental illness suffered from a comorbid SUD, although comorbidity estimates range between 9 and 95% according to a recent meta-analysis (Fazel and Seewald, 2012; Baranyi et al., 2022).

With regard to Spain, which has one of the largest prison population rates compared to the European median (Aebi and Tiago, 2021), only a single study has investigated the prevalence of psychiatric disorders among male prisoners so far (Vicens et al., 2011). The lifetime and last month prevalence of mental disorders was 84.4 and 41.2%, respectively, including SUD (76.2 vs. 17.5%), anxiety disorder (45.3 vs. 23.3%), mood disorder (41.0 vs. 14.9%), and psychotic disorder (10.7 vs. 4.2%) among the most frequent ones (Vicens et al., 2011). On the other hand, over 80% of the men had at least one personality disorder, which was commonly a cluster B personality disorder, although the rate of antisocial personality disorder (23.0%) was lower than that reported in the literature (47.0%) (Fazel and Danesh, 2002; Vicens et al., 2011). Importantly, the authors did not evaluate ADHD, despite the elevated rates consistently observed among juvenile offenders worldwide, the evidence that symptoms persist into adulthood for most children, and the high comorbidity with other mental disorders (Haavik et al., 2010; Young et al., 2015; Solberg et al., 2018). Likewise, numerous studies report a notable overrepresentation of other neurodevelopmental disabilities, such as dyslexia, among detained adolescents and incarcerated young people but the prevalence of learning disorders in Spanish inmates is not available (Einat and Einat, 2008; Elbeheri et al., 2009; Hughes et al., 2012; Borschmann et al., 2020). Besides, there are no reliable data on comorbidity within Spanish prisons, since Vicens et al. (2011) focused on male inmates with co-occurring SUD and Axis I psychiatric disorders (Vicens et al., 2011).

Previous epidemiological investigations, however, have reported a wide variability in prevalence rates (Fazel and Danesh, 2002; Fazel and Seewald, 2012; Baranyi et al., 2022; Heller et al., 2022). This heterogeneity may reflect distinct research methodologies, diagnostic criteria, assessment tools, sample characteristics, and information sources (Young and Cocallis, 2019). For instance, most of the research has relied on screening measures, medical records or self-reports, which might have yielded unreliable estimates (Martin et al., 2013). Thus, there is a call for the use of standardized clinical interviews based on the Diagnostic and Statistical Manual of Mental Disorders (DSM) or the International Classification of Diseases (ICD) criteria to improve the diagnostic validity (Slade and Forrester, 2013; Hofvander et al., 2017). Indeed, accurate prevalence estimates are crucial for planning and implementing appropriate rehabilitation services during imprisonment. Yet, to the best of our knowledge, the prevalence of mental disorders and comorbidities among offenders completing prison sentences has never been examined. Given that the majority do not receive adequate mental health care during incarceration (Bowler et al., 2018), we hypothesize that inmates who are about to be released are still characterized by a high rate of psychiatric disorders, which can undermine the effectiveness of vocational programs aimed at promoting their successful reintegration into society.

Considering this knowledge gap and the above-mentioned weaknesses of prior studies, the present research used standardized diagnostic methods based on the DSM criteria to evaluate the prevalence of current and lifetime mental disorders among 140 inmates (93.6% male, 39.5 ± 10.4 years) at the end of their sentence who participated in an occupational program. Moreover, we aimed to determine, for the first time in Spain, the comorbidity rates within this adult prison population and whether specific mental disorders were related to a higher risk of repeat incarceration or violent crimes. In this sense, previous investigations from other countries suggest that personality and neurodevelopmental disorders (i.e., ADHD, reading problems) are positively related to violent offenses, while subjects with SUD, depression, or generalized anxiety disorder are more likely to be incarcerated for a non-violent crime (Lewis et al., 1980; Lindgren et al., 2002; Simonoff et al., 2004; Harris et al., 2006; Macciò et al., 2015; Alevizopoulos and Igoumenou, 2016; Scott et al., 2016; Román-Ithier et al., 2017; Nacher et al., 2018). On the other hand, antisocial personality disorder, SUD, bipolar disorder, depression, psychotic disorders, and ADHD have been significantly associated with recidivism, especially when co-occurring disorders are being present (Baillargeon et al., 2009, 2010; Yu et al., 2012; Gaïffas et al., 2014; Macciò et al., 2015; Mohr-Jensen and Steinhausen, 2016; Román-Ithier et al., 2017; Philipp-Wiegmann et al., 2018).

## Materials and methods

### Study population

Participants were consecutively recruited over a 1-year period from an occupational program aimed at promoting the fully integration into society of inmates who are ending their sentence (Reincorpora Program; Fundació “la Caixa”). The program was restricted to subjects with a valid Spanish ID card or an employment authorization for offenders, who indicated their willingness to initiate the rehabilitation process and were at the lowest category within the prison system, which allows day release privileges. Otherwise, they had to be on parole or convicted with alternative measures to imprisonment. Eligible candidates were required to be at least 18 years and have sufficient language skills to give informed consent and complete the clinical assessment. The participation was totally voluntary at all times and prisoners were reassured that their personal details would not be identifiable. Of the 179 subjects who met inclusion criteria, 39 (21.8%) refused to participate, which yielded a study sample of 140 (78.2%) inmates.

### Procedure

The present study was conducted in cooperation with the Department of Justice of the Catalan Government and the Center for Reintegration Initiatives (CIRE), a public enterprise that facilitates the social integration of inmates through training programs and job opportunities. After obtaining authorization from the directors of the centers where subjects were serving their sentence, all potential participants were informed about the nature and purpose of the study. Only those who provided their informed consent were interviewed for the assessment of mental disorders with the Structured Clinical Interview for DSM-IV Axis I and II Disorders (SCID-I and SCID-II) (First et al., 1997a,b), the Diagnostic Interview for ADHD in adults (DIVA 2.0) (Kooij, 2012), the Battery for the Evaluation of Reading Processes in Junior and Senior High-School Students, Revised (PROLEC-SE-R) (Cuetos et al., 2016), and the Battery for the Evaluation of Writing Processes (PROESC) (Cuetos et al., 2002). Interviews were carried out during three sessions in the CIRE facilities, where the inmates received the occupational program. A suitable place was arranged so that psychiatrists and psychologists from the Hospital Universitari Vall d’Hebron, in Barcelona, could perform the clinical assessment away from the distraction of other ongoing activities. Participants first met a psychiatrist of the research team, who conducted a clinical interview including the SCID-I, SCID-II, and DIVA 2.0, and were then granted an additional appointment with a psychologist who administered the neuropsychological battery for the evaluation of learning disorders. All researchers had extensive experience in the administration of the instruments used as they are part of the assessment protocol from the Adult ADHD Program at the Department of Psychiatry, and did not require special training to perform the diagnostic interviews. The study protocol received formal approvals from the Ethics Committee of the Vall d’Hebron Hospital Universitari, the Department of Justice, and the involved penitentiary centers.

### Measures

#### Sociodemographic and criminal characteristics

The CIRE provided information on the sociodemographic and criminal characteristics of prisoners who took part of the study. It included gender (men, women), age, nationality, civil status (single, in a relationship), educational level (less than compulsory education, compulsory education or higher), employment situation prior to imprisonment (unemployed, employed), age of first conviction, number of imprisonments, reason for incarceration, and length of stay. Based on the type of offense leading to incarceration, participants were classified in two major crime categories: violent crimes and non-violent crimes. Violent crimes included robbery with violence, property crimes with violence, bodily harm, domestic violence, organized crime, attempted homicide, and murder. Non-violent crimes were defined as robbery, vandalism, traffic offenses, tax evasion, computer crimes, obstruction of justice, parole violation, drug possession, and trafficking.

#### Psychiatric disorders

Current and past psychiatric disorders were determined with the Spanish version of the Structured Clinical Interview for DSM-IV (SCID-I and SCID-II), a semi-structured diagnostic interview that assesses Axis I and Axis II disorders according to the DSM-IV criteria (First et al., 1997a,b). The procedure consists of an open-ended overview that includes questions about demographic information, past and present periods of psychopathology, treatment history and current functioning, followed by separate diagnostic modules. The Diagnostic Interview for ADHD in adults (DIVA 2.0), which is available and validated in Spanish, was administered to diagnose ADHD, both at current time (adulthood) and retrospectively (childhood, before the age of seven) (Kooij, 2012; Ramos-Quiroga et al., 2019). It evaluates the presence of ADHD symptoms as reported in the DSM-IV and the impairment caused in five areas of daily life (i.e., work and education, relationships and family life, social contacts, free time and hobbies, self-confidence, and self-image) by providing several specific examples, for each age period, to facilitate recognition. Additionally, learning disorders were examined with the Battery for the Evaluation of Reading Processes in Junior and Senior High-School Students, Revised (PROLEC-SE-R) (Cuetos et al., 2016) and the Battery for the Evaluation of Writing Processes (PROESC) (Cuetos et al., 2002).

The most prevalent psychiatric disorders were grouped into broader categories for analyses: Mood disorders (i.e., major depressive disorder, mood disorder due to a general medical condition, and substance-induced mood disorder), psychotic disorders (i.e., schizophrenia, delusional disorder, and substance-induced psychotic disorder), SUD (i.e., alcohol, hallucinogens, amphetamines, cannabis, cocaine, opioids, sedatives, and other substances), anxiety disorders (i.e., social phobia, post-traumatic stress disorder, generalized anxiety disorder, substance-induced anxiety disorder, and anxiety disorder not otherwise specified), ADHD, learning, and personality disorders (i.e., paranoid, schizoid, antisocial, borderline, narcissistic, avoidant, dependent, and obsessive-compulsive).

### Statistical analyses

All analyses were performed in SPSS version 25.0 (IBM), with a two-tailed *p*-value ≤ 0.05 as significance threshold. Descriptive statistics were calculated to summarize the sociodemographic and criminal characteristics of prisoners, and to estimate the prevalence rates of mental disorders. Multivariate regression analyses were then performed to identify significant variables for repeat incarceration (continuous variable) and violent offending (yes, no). Potential predictors included factors which have been previously addressed in the literature (Lewis et al., 1980; Lindgren et al., 2002; Harris et al., 2006; Macciò et al., 2015; Alevizopoulos and Igoumenou, 2016; White et al., 2016; Kim et al., 2017; Krona et al., 2017; Román-Ithier et al., 2017; Garofalo et al., 2018; Nacher et al., 2018; Fernández-Pacheco Alises et al., 2022; Karlsson and Håkansson, 2022; Streb et al., 2022); namely, gender (men, women), age (continuous variable), nationality (Spanish, foreign origin), educational level (less than compulsory education, compulsory education or higher), number of lifetime diagnoses (continuous variable), history of any mood disorder (yes, no), psychotic disorder (yes, no), SUD (yes, no), anxiety disorder (yes, no), and ADHD (yes, no), any current learning disorder (yes, no), and personality disorder (yes, no). However, given its limited sample size, the present study was not sufficiently powered to test all potential predictors simultaneously. Therefore, bivariate analyses were first conducted, using Fisher’s exact tests, Chi-square tests, Student’s *t*-tests or Pearson’s correlation coefficients, and variables with *p*-values < 0.1 were subsequently entered in the final models, as described in previous investigations (Fazel and Seewald, 2012; Kim et al., 2017). Last, we undertook sensitivity analyses by entering all potential predictors in the models to assess the robustness of the findings and minimize the instances of *p*-hacking (Raj et al., 2018).

## Results

### Sample characteristics

The sample included 131 (93.6%) men and 9 women (6.43%), with ages ranging from 20 to 66 years (*M* = 39.5, *SD* = 10.1). Most of the participants (87.9%) completed compulsory education or had a higher degree, 55.1% had been employed prior to incarceration, and 45% were in a relationship. Subjects were predominantly Spanish (61.4%) and those of foreign origin (38.6%) came mostly from Spanish-speaking countries (20.0%) and Morocco (11.4%). Approximately 60% of the inmates were sentenced for a violent crime (e.g., robbery with violence, domestic violence, bodily harm, and murder) and 43 (30.7%) had already been incarcerated for previous convictions, including at least one violent act in most cases. Overall, the average number of imprisonments varied from 1 to 12 (*M* = 1.62, *SD* = 1.47) and the age of first conviction was 30.6 years (*SD* = 11.0, range = 16–59). Table 1 presents the sociodemographic and criminal characteristics of the sample.

**TABLE 1:** Sociodemographic and criminal characteristics of the study population.

	Total sample (*n* = 140)
**Sociodemographic characteristics**	
Gender (*n*, %)	
Men	131 (93.6)
Women	9 (6.43)
Age, years (*M*, *SD*)	39.5 (10.1)
Nationality (*n*, %)	
Spain	86 (61.4)
Europe	7 (5.00)
Latin America	28 (20.0)
Africa	17 (12.1)
Asia	2 (1.43)
Civil status (*n*, %)	
Single	77 (55.0)
Married or with a partner	63 (45.0)
Education level (*n*, %)	
Less than compulsory education	17 (12.1)
Compulsory education or higher	123 (87.9)
Employment status prior to imprisonment (*n*, %)	
Unemployed	31 (22.5)
Employed	76 (55.1)
Other	31 (22.5)
**Criminal record**	
Age of first conviction (*M*, *SD*)	30.6 (11.0)
Previous imprisonment (*n*, %)	
No	97 (69.3)
Yes	43 (30.7)
Number of imprisonments (*M*, *SD*)	1.62 (1.47)
Length of last imprisonment, months (*M*, *SD*)	79.5 (65.2)
Type of offenses (*n*, %)	
Robbery with violence	44 (31.7)
Property crimes with violence	3 (2.16)
Bodily harm	9 (6.47)
Domestic violence	10 (7.19)
Organized crime, terrorist organization	4 (2.88)
Attempted homicide	4 (2.88)
Murder	9 (6.47)
Robbery	9 (6.47)
Vandalism	1 (0.72)
Traffic offenses	3 (2.16)
Tax evasion, fraud	8 (5.76)
Computer crimes	1 (0.72)
Obstruction of justice	1 (0.72)
Parole violation	2 (1.44)
Drug possession	20 (14.4)
Drug trafficking	11 (7.91)

Differences in sample sizes across variables are due to missing data.

### Lifetime prevalence of mental disorders and comorbidities

A large majority of prisoners (81.4%) met criteria for at least one Axis I disorder according to the DSM-IV. The most common diagnoses were SUD (51.4%), followed by ADHD (31.4%) (Table 2). In particular, subjects suffering from SUD mainly had problems with cocaine (32.1%), alcohol (27.9%), and cannabis (18.6%) misuse, and most of them abused multiple substances during their lifetime. With regard to ADHD, a total of 21 (15.0%) participants were diagnosed with the combined subtype, 8.57% met criteria for the hyperactive-impulsive subtype, and 7.86% had the inattentive subtype. Furthermore, 24 (17.1%) prisoners were found to meet criteria for a lifetime psychotic disorder, mostly induced by substance use (15.7%), while 13.6% suffered from internalizing disorders, including any anxiety and mood disorder (7.14 and 6.43%, respectively) (Table 2).

**TABLE 2:** Lifetime and current prevalence of mental disorders.

	Lifetime prevalence % (*n*/*N*)	Current prevalence % (*n*/*N*)
Axis I disorders	81.4 (105/129)	59.0 (72/122)
Mood disorders	6.43 (9/140)	1.43 (2/140)
Major depressive disorder	4.29 (6/140)	1.43 (2/140)
Mood disorder due to a general medical condition	0.71 (1/140)	0.00 (0/140)
Substance-induced mood disorder	2.14 (3/140)	0.71 (1/140)
Substance use disorders (dependence/abuse)	51.4 (72/140)	5.71 (8/140)
Alcohol	27.9 (39/140)	2.14 (3/140)
Hallucinogens	3.57 (5/140)	0.00 (0/140)
Amphetamines	4.29 (6/140)	0.00 (0/140)
Cannabis	18.6 (26/140)	3.57 (5/140)
Cocaine	32.1 (45/140)	0.71 (1/140)
Opioids	15.0 (21/140)	0.00 (0/140)
Sedatives	3.57 (5/140)	1.43 (2/140)
Other substances	0.71 (1/140)	0.00 (0/140)
Psychotic disorders	17.1 (24/140)	0.71 (1/140)
Schizophrenia	0.71 (1/140)	0.71 (1/140)
Delusional disorder	0.71 (1/140)	0.00 (0/140)
Substance-induced psychotic disorder	15.7 (22/140)	0.00 (0/140)
Anxiety disorders	7.14 (10/140)	2.86 (4/140)
Social phobia	0.71 (1/140)	0.71 (1/140)
Post-traumatic stress disorder	0.71 (1/140)	0.00 (0/140)
Generalized anxiety disorder	1.43 (2/140)	1.43 (2/140)
Substance-induced anxiety disorder	1.43 (2/140)	0.00 (0/140)
Anxiety disorder NOS	2.86 (4/140)	0.71 (1/140)
Attention deficit/hyperactivity disorder	31.4 (44/140)	16.4 (23/140)
Inattentive	7.86 (11/140)	3.57 (5/140)
Hyperactive-impulsive	8.57 (12/140)	7.86 (11/140)
Combined	15.0 (21/140)	5.00 (7/140)
Learning disorders	NA	38.6 (54/120)
Reading		6.67 (8/120)
Written expression		16.7 (20/120)
Reading and written expression		21.7 (26/120)
Axis II disorders	NA	26.5 (36/136)
Cluster A		
Paranoid personality disorder		4.41 (6/136)
Schizoid personality disorder		1.47 (2/136)
Cluster B		
Antisocial personality disorder		12.5 (17/136)
Borderline personality disorder		0.74 (1/136)
Narcissistic personality disorder		0.74 (1/136)
Cluster C		
Avoidant personality disorder		1.47 (2/136)
Dependent personality disorder		0.74 (1/136)
Obsessive-compulsive personality disorder		4.41 (6/136)
Comorbid disorders		
No mental disorder	13.3 (16/120)	28.3 (34/120)
One mental disorder	25.8 (31/120)	48.3 (58/120)
Two mental disorders	33.3 (40/120)	18.3 (22/120)
Three mental disorders	15.8 (19/120)	5.00 (6/120)
Four or more mental disorders	11.7 (14/120)	0.00 (0/120)

Differences in sample sizes across variables are due to missing data. NA, not applicable; NOS, not otherwise specified.

As shown in Table 2, there was a high rate of comorbidity (60.8%), ranging from two to five disorders. Specifically, 33.3% of the inmates were diagnosed with two mental illnesses during their lifetime, 15.8% had three diagnoses, and 11.7 met criteria for at least four disorders.

### Current prevalence of mental disorders and comorbidities

Axis I disorders were currently present in 59.0% of the sample, including learning disorders (38.6%), ADHD (16.4%), and SUD (5.71%) among the most frequent clinical syndromes (Table 2). Particularly, almost half of the inmates who were diagnosed with a learning disorder had difficulties in reading and written expression, which represented 21.7% of the entire sample. Eleven (7.86%) participants suffered from the ADHD hyperactive-impulsive subtype, 7 (5.00%) met criteria for the combined subtype, and 5 (3.57) had the inattentive subtype. With regard to SUD, cannabis- and alcohol-related disorders were the most prevalent categories (3.57 and 2.14%, respectively). A current psychotic disorder was only found in one (0.71%) subject, who received a diagnosis of schizophrenia. Anxiety disorders were detected in four (2.86%) prisoners, half of whom had generalized anxiety disorder, and the prevalence rate of mood disorders (i.e., major depressive disorder, substance-induced mood disorder) was 1.43% (Table 2).

Moreover, 36 (26.5%) participants met criteria for a current Axis II disorder, which commonly was an antisocial personality disorder (12.5%), a paranoid personality disorder, or an obsessive-compulsive personality disorder (both 4.41%) (Table 2).

Overall, 48.3% of the inmates had only one mental illness, 18.3% were diagnosed with two psychiatric disorders, and 5.00% suffered from three comorbid disorders.

### Association between psychiatric disorders and criminal history

Bivariate analyses indicated that repeat incarceration was significantly associated with age, nationality, and comorbidity. Specifically, the number of imprisonments was higher among older inmates (*r*_(139)_ = 0.225, *p* = 0.008), Spanish (1.91 vs. 1.17, *t*_(138)_ = 2.98, *p* = 0.003), and subjects who received multiple diagnoses during their lifetime (*r*_(120)_ = 0.253, *p* = 0.005). The influence of nationality did not remain significant (*p* = 0.134) in the multivariate linear regression model, whereas age (β = 0.039, *p* = 0.005) and comorbidity (β = 0.28, *p* = 0.016) increased the number of imprisonments, even when all potential predictors were considered (age: β = 0.037, *p* = 0.007; comorbidity: β = 0.26, *p* = 0.029). Furthermore, when we examined the main diagnostic categories, we found a significant relationship between the number of imprisonments, ADHD (2.18 vs. 1.36, *t*_(138)_ = −3.15, *p* = 0.002), and SUD (2.00 vs. 1.22, *t*_(138)_ = −3.24, *p* = 0.002), even after controlling for age and nationality. Indeed, prisoners with a lifetime diagnosis of ADHD or SUD had higher odds of being in jail more than once (Table 3). Mood disorders, psychotic disorders, anxiety disorders, learning disorders, and personality disorders, by contrast, showed no link with repeat incarceration (*p* > 0.1). Sensitivity analysis showed consistent results, indicating the robustness of these findings (age: β = 0.041, *p* = 0.003; SUD: β = 0.68, *p* = 0.026; ADHD: β = 0.64, *p* = 0.034).

**TABLE 3:** Variables associated with repeat incarceration in the multivariate linear regression analysis.

Independent variables	*β*	*SE*	*P*-value
Age	0.031	0.012	0.008
Nationality	−0.23	0.27	0.403
Lifetime SUD	0.54	0.25	0.034
Lifetime ADHD	0.70	0.26	0.009

ADHD, attention deficit/hyperactivity disorder; *SE*, standard error; SUD, substance use disorders.

On the other hand, inmates convicted of at least one violent act were more frequently men (97.7 vs. 2.30%, χ^2^ (1, *N* = 140) = 6.52, *p* = 0.027) and met criteria for more lifetime psychiatric disorders (2.18 vs. 1.43, *t*_(118)_ = −3.23, *p* = 0.002), according to bivariate analyses. These associations remained significant when both factors were taken into account [men: odds ratio (*OR*) = 10.2, 95% confidence interval (*CI*) = 1.18–88.1, *p* = 0.035; comorbidities: *OR* = 1.59, 95% *CI* = 1.12–2.27, *p* = 0.010] and were not affected by the adjustment for all potential covariates (men: *OR* = 13.1, 95% *CI* = 1.39–122.9, *p* = 0.024; comorbidities: *OR* = 1.54, 95% *CI* = 1.05–2.26, *p* = 0.027). Regarding the main diagnostic categories, only SUD [63.2 vs. 32.1%, χ^2^ (1, *N* = 140) = 12.8, *p* < 0.001], ADHD [36.8 vs. 22.6%, χ^2^ (1, *N* = 140) = 3.06, *p* = 0.080], and learning disorders [53.9 vs. 29.5%, χ^2^ (1, *N* = 120) = 6.70, *p* = 0.010] were overrepresented among violent offenders. In the adjusted model, significant predictors included gender, having a lifetime diagnosis of SUD and current learning disorders, while ADHD was no longer associated with violent offending (Table 4). These results seem to be robust, since the risk estimates hardly changed with sensitivity analysis (men: *OR* = 14.7, 95% *CI* = 1.36–159.2, *p* = 0.027; SUD: *OR* = 4.05, 95% *CI* = 1.50–10.9, *p* = 0.006; learning disorders: *OR* = 2.63, 95% *CI* = 1.02–6.76, *p* = 0.045).

**TABLE 4:** Variables associated with violent offending in the multivariate logistic regression analysis.

Independent variables	*OR*	95% *CI*	*P*-value
Gender (ref. women)	10.3	1.08–98.0	0.043
Lifetime SUD (ref. no)	3.39	1.45–7.95	0.005
Lifetime ADHD (ref. no)	1.68	0.67–4.22	0.274
Current LD (ref. no)	2.69	1.13–6.36	0.025

ADHD, attention deficit/hyperactivity disorder; *CI*, confidence interval; LD, learning disorders; *OR*, odds ratio; SUD, substance use disorders.

## Discussion

The current research investigates for the first time the prevalence of mental disorders and comorbidities in Spanish inmates who are about to be released, and their association with repeat incarceration and violent offending. Using standardized diagnostic instruments, our findings demonstrate that prisoners at the end of their sentence are still characterized by a high prevalence of psychiatric disorders, which can significantly jeopardize the effectiveness of occupational programs aimed at promoting their successful reintegration into society. Specifically, almost half of the participants (48.3%) met criteria for a current Axis I or Axis II disorder and 23.3% suffered from two or more comorbid disorders, including learning disorders, personality disorders, ADHD, and SUD among the most common diagnoses. In addition, the present study showed that older age and a history of ADHD increased the number of imprisonments. On the other hand, inmates convicted of at least one violent crime during their lifetime were more likely to be men and present a current learning disorder. Finally, having a lifetime diagnosis of SUD or multiple psychiatric disorders appeared to be associated with both repeat incarceration and violent offending.

Overall, the results confirmed the high prevalence of psychiatric disorders previously identified among prisoners across different countries, although estimates have been shown to vary widely (e.g., 41–90%) (Vicens et al., 2011; Macciò et al., 2015; Alevizopoulos and Igoumenou, 2016; Garofalo et al., 2018; Nacher et al., 2018; Heller et al., 2022). Of note, the only previous study conducted in Spanish male prisoners reported a 1-month prevalence of 41.2% for any Axis I disorder according to the SCID-I (Vicens et al., 2011), which is particularly close to our figures (i.e., 59%). More specific, SUD was one of the most frequent diagnoses (17.5%) and 76.2% of the inmates appeared to have a history of drug misuse (abuse and/or dependency), with cocaine, cannabis, and alcohol being the primarily consumed substances (Vicens et al., 2011). Likewise, SUD were highly overrepresented in the present study, where 72 (51.4%) subjects received a lifetime diagnosis, and the most commonly used drug was cocaine, followed by alcohol and cannabis. The current prevalence of SUD, on the other hand, fell to 5.71%, which could be partly explained by the institutional bans and detoxification programs implemented in prison. Indeed, a considerable body of evidence suggests that imprisonment may represent a unique opportunity to identify individuals with a history of substance use and initiate addiction treatments that significantly improve their health (Degenhardt et al., 2014; de Andrade and Kinner, 2016; Mundt et al., 2018; Bukten et al., 2020). However, the majority of interventions fail to produce long-term benefits and most prisoners return to use substances upon release (Puljević et al., 2017). In this sense, it should be noted that while the observed current prevalence was considerably low compared to lifetime prevalence, SUD were still five times more frequent among prisoners who were ending their sentence than in the general Spanish population (Navarro-Mateu et al., 2015). Moreover, our research showed that having a history of SUD was related to repeat incarceration and increased the risk of being convicted for a violent crime, which is consistent with previous studies (Steadman et al., 1998; Baillargeon et al., 2010; Young et al., 2011; Macciò et al., 2015; White et al., 2016; Nacher et al., 2018). Taken together, the present findings emphasize the need to promote detoxification treatments both in prison and as individuals with SUD transition back to the community in order to facilitate their reintegration and employability (Bukten et al., 2020). Indeed, Bahr et al. (2010) examined the extent to which drug treatment was associated with the reentry of parolees after release and found that those who succeeded were more likely to have taken a substance abuse class while in prison (Bahr et al., 2010).

On the other hand, the prevalence of personality, anxiety, mood, and psychotic disorders in our sample contrasts starkly with estimates reported by Vicens et al. (2011). Particularly, these authors found that personality, anxiety, mood, and psychotic disorders were among the most common diagnoses, with a 1-month prevalence of 82.3, 23.3, 14.9, and 4.2%, respectively (Vicens et al., 2011). Nevertheless, only 36 (26.5%) of our participants met criteria for a current Axis II disorder, four (2.86%) suffered from anxiety disorders, and mood disorders were present in two (1.43%) inmates. Finally, although considerably lower, our rates of psychotic disorders (i.e., 0.71%) are grossly elevated compared with those within the community, where the prevalence for schizophrenia and related disorders is estimated to be 0.4% (Moreno-Küstner et al., 2018), and echo the values provided by Macciò et al. (2015) in Italian prisoners (i.e., 1.3%). Likewise, Bulten et al. (2009) showed that only one (0.5%) of 191 inmates admitted to the general ward of a Dutch correctional institution was diagnosed with such disorders. In contrast, the lifetime prevalence for psychotic disorders in the present study was 17.1%, most of whom (91.7%) were substance-induced, which confirms that substance use is a factor increasing the chance of incarceration (Baillargeon et al., 2010) and demonstrates the effectiveness of the prison system in detecting and monitoring drug users.

Interestingly, Vicens et al. (2011) did not evaluate ADHD, despite the elevated rates consistently observed among juvenile offenders worldwide and the evidence that symptoms persist into adulthood for most children. Indeed, a meta-analysis based on 42 studies provided a prevalence of 25.5% among youth and adult incarcerated populations when using diagnostic clinical interviews (Young et al., 2015). More recently, Vélez-Pastrana et al. (2020) conducted a cross-sectional study in 500 Latino male prisoners aged 18–74 and found that 17% met DSM diagnostic criteria for adult ADHD. Similarly, Hamzeloo et al. (2016) reported a prevalence of 16.2% among adult male inmates in Iran. Consistent with these previous investigations, 23 (16.4%) of our participants suffered from ADHD, which is substantially higher than the rate detected in adult community samples (i.e., 2.5–4.4%) (Kessler et al., 2006; Fayyad et al., 2007; Simon et al., 2009). Besides, 44 (31.4%) subjects received a lifetime diagnosis of ADHD and childhood ADHD was found to be associated with a greater number of imprisonments as described elsewhere (Gaïffas et al., 2014; Mohr-Jensen and Steinhausen, 2016; Román-Ithier et al., 2017; Philipp-Wiegmann et al., 2018). Research also suggests that ADHD may negatively impact rehabilitation and educational programs, and inmates with ADHD are more likely to exhibit poorer outcomes following release (Cahill et al., 2012; Scott et al., 2016). Specifically, according to longitudinal studies subjects who present the hyperactive-impulsive subtype, which was the most currently frequent in the present sample (i.e., 7.86%), may be at greater risk of ending up in prison as a result of behaviors such as fast driving, illegal drug use, impulsive gambling, and a variety of antisocial activities (Barkley et al., 2004; Satterfield et al., 2007; Cahill et al., 2012). Given the disproportionately high rate of ADHD still present among prisoners who are in the final stage of their sentence and the increased probability of further incarcerations, our results highlight that providing appropriate mental health care within the prison setting and after release is crucial to reduce recidivism, enhance the rehabilitation process of inmates, and facilitate their successful integration in the community. In this vein, effective management of ADHD has shown to improve psychiatric symptoms and comorbidities, decrease violent and delinquent behavior, increase participation in social, vocational, and/or educational programs, and promote employability after release (Scott et al., 2016).

Furthermore, there is significant evidence that a high proportion of adults with ADHD may also have a comorbid condition, including other neurodevelopmental disorders. However, studies examining the prevalence of such disabilities within the prison system are still limited. Therefore, one salient finding of the current research concerns the high frequency of learning disorders identified among inmates who are about to be released. In particular, the rate of learning disorders was 38.6%, with reading difficulties being present in 28.3% of the sample, while the prevalence for dyslexia in the general population stands at 10% (Hughes et al., 2012). In accordance with our results, previous investigations from several Western criminal justice systems have reported a notable overrepresentation of learning disorders among incarcerated adults, although estimates strongly vary (e.g., 30–76%) as a result of differences in the definitions and assessment procedures used (Einat and Einat, 2008; Elbeheri et al., 2009; Hughes et al., 2012). Indeed, research that identifies disproportionately large rates of dyslexia within prisons may be confounding specific learning difficulties with more generalized learning disabilities derived from low intelligence (Elbeheri et al., 2009). On the other hand, this study revealed that inmates convicted of at least one violent crime during their lifetime were more likely to present a learning disorder, which provides further evidence for the contribution of these disabilities to criminality. Similarly, reading problems have been found to be associated with antisocial or aggressive behaviors, violence, and recidivism (Lewis et al., 1980; Lindgren et al., 2002; Simonoff et al., 2004; Harris et al., 2006). As learning difficulties manifest early in life, subjects whose dyslexia goes undiagnosed or receive inappropriate educational support might experience considerable pain, social stigma, harassment, and denigration in the school environment, which might turn into forms of deviant behavior as a way of responding to their low self-esteem and achieve peer recognition (Elbeheri et al., 2009). Besides, this environment may discourage many people with such problems from attending school and eventually lead to drop-out, thus increasing the risk of engagement in criminal activity as suggested by earlier studies that indicate a causal relationship between quitting school, lack of education, and offending behavior (Einat and Einat, 2008). Finally, language impairments are common among adults with learning disabilities (Vogel, 1997) and poor communication skills may result in difficulties understanding the perspective of others or the use of challenging behavior, including reactive aggression, as a means to express feelings (Hughes et al., 2017).

Overall, the significant prevalence of neurodevelopmental disorders found among prisoners who are ending their sentence questions the effectiveness of the practices used within the justice system to identify and support these vulnerable individuals. For instance, current approaches tend to assume typical levels of verbal and cognitive competence, and may be inappropriate for people with such conditions given their specific developmental needs and learning styles. Thus, a lack of identification or insufficient awareness of the varied needs associated with neurodevelopmental disorders can lead to poor engagement in interventions intended to address offending behavior, promote rehabilitation, and increase employability (Hughes et al., 2017; Borschmann et al., 2020). Conversely, programs that recognize the difficulties of the individual typically yield better learner achievement (Reid and Kirk, 2001).

Finally, another key finding was related to psychiatric comorbidities, given the sparse research and wide variability in prevalence rates observed across countries and specific settings. In this sense, previous investigations, including that from Vicens et al. (2011), have mainly examined co-occurring SUD and Axis I disorders, with estimates ranging between 6 and 95% (Vicens et al., 2011; Fazel and Seewald, 2012; Baranyi et al., 2022). Of note, 60.8% of the participants had multiple psychiatric disorders during their lifetime and 23.3% were currently diagnosed with two or more comorbid conditions, suggesting that comorbidity represents more a rule than an exception among the incarcerated population (Bebbington et al., 2017; Garofalo et al., 2018; Heller et al., 2022). Furthermore, our study revealed that the risk for repeat incarceration and violent offending increased with the number of lifetime diagnoses. Consistently, the presence of psychiatric comorbidities has been related to less social competence, worse treatment outcomes, and higher odds of relapse into crime, especially violent reoffending (Chang et al., 2015; Kim et al., 2017; van Buitenen et al., 2020).

### Strengths and limitations

The findings of this first attempt to estimate the prevalence of mental disorders and comorbidities among offenders completing prison sentences should be interpreted in light of several limitations. First, the assessment of past disorder symptoms based on participants’ recall might have undercounted lifetime prevalence, since we were unable to corroborate this information with collateral reports or medical records (Navarro-Mateu et al., 2015). However, the semi-structured design of the diagnostic instruments used, which were administered by experienced clinicians, have shown to diminish this recall bias (Knäuper et al., 1999). Some potential confounders, including life events, financial problems or parental psychopathology, were not available either. Therefore, the retrospective and cross-sectional design of the study prevents from establishing conclusions on causality between variables, although retrospective studies may be an important tool to investigate rare phenotypes, such as repeat incarceration or violent offending, on relatively large samples from neurodevelopmental and lifetime mental health perspectives (Hofvander et al., 2017; Talari and Goyal, 2020). Besides, the relatively small sample size may have limited the power of our analyses to determine additional significant correlations. Lastly, participants were recruited from an occupational program aimed at promoting the integration into society of inmates. In particular, the program was restricted to subjects with a valid Spanish ID card or an employment authorization for offenders, who indicated their willingness to initiate the rehabilitation process and were at the lowest category within the prison system, which allows day release privileges. Otherwise, eligible candidates had to be on parole or convicted with alternative measures to imprisonment. Therefore, findings are not representative of the entire Spanish prison population and may not generalize to other regions or samples. By contrast, the main strengths include the involvement of women as study participants, the availability of official offending history from criminal records, and the use of standardized clinical interviews based on the DSM criteria, such as SCID-I and SCID-II, which leads to more replicable and valid diagnoses (Segal et al., 2012). Nevertheless, the SCID does not provide information on the severity of the evaluated disorders and some psychiatric diagnoses (e.g., intellectual disabilities, autism spectrum disorders, oppositional defiant disorder, sexual disorders) are not covered. Finally, the assessment of both lifetime and current prevalence gives a wider view of the mental health problems among incarcerated adults.

## Conclusion

Our results demonstrate that inmates who are about to be released are still characterized by a higher rate of psychiatric disorders than the general population, including neurodevelopmental disorders and SUD among the most common diagnoses. On the other hand, childhood ADHD was found to be associated with a greater number of imprisonments and subjects convicted for a violent crime were more likely to present a learning disorder, while a history of SUD was related to both repeat incarceration and violent offending. Given the increased risk of recidivism among mentally ill inmates and the challenges they confront to benefit from occupational programs, the present research highlights the need for early identification and provision of appropriate psychiatric care within the prison setting and after release in order to facilitate their successful reintegration in the community. However, further epidemiological and longitudinal studies, with larger representative samples, are required to provide accurate prevalence estimates and fully validate our results. Future research should also determine whether rehabilitation services tailored to fit the abilities, learning styles, and needs of offenders with mental disorders can ultimately change their trajectory and improve outcomes following release.

## Data availability statement

The raw data supporting the conclusions of this article will be made available by the authors, without undue reservation.

## Ethics statement

The studies involving human participants were reviewed and approved by the Ethics Committee of the Vall d’Hebron Hospital Universitari, the Department of Justice (Generalitat de Catalunya, Spain), and the involved penitentiary centers. The patients/participants provided their written informed consent to participate in this study.

## Author contributions

MP and SV: study conception and design, data analysis, and interpretation of results. LD: data collection. SV: data curation. RB and MC: study supervision. MC: funding acquisition. MP: writing—draft manuscript preparation. SV, RB, and MC: writing—review and editing. All authors read and approved the final version of the manuscript.

## References

[B1] Aebi M. F. Tiago M. M. (2021). *Prisons and prisoners in Europe 2020: Key findings of the SPACE I report.* Strasbourg: Council of Europe and University of Lausanne.

[B2] Alevizopoulos G. Igoumenou A. (2016). Psychiatric disorders and criminal history in male prisoners in Greece. *Int. J. Law Psychiatry* 47 171–175. 10.1016/j.ijlp.2016.04.003 27167215

[B3] Bahr S. J. Harris L. Fisher J. K. Harker Armstrong A. (2010). Successful reentry: What differentiates successful and unsuccessful parolees? *Int. J. Offender Ther. Comp. Criminol.* 54 667–692. 10.1177/0306624X09342435 19638473

[B4] Baillargeon J. Binswanger I. A. Penn J. V. Williams B. A. Murray O. J. (2009). Psychiatric disorders and repeat incarcerations: The revolving prison door. *Am. J. Psychiatry* 166 103–109. 10.1176/appi.ajp.2008.08030416 19047321

[B5] Baillargeon J. Penn J. V. Knight K. Harzke A. J. Baillargeon G. Becker E. A. (2010). Risk of reincarceration among prisoners with co-occurring severe mental illness and substance use disorders. *Adm. Policy Ment. Health* 37 367–374. 10.1007/s10488-009-0252-9 19847638

[B6] Baranyi G. Fazel S. Langerfeldt S. D. Mundt A. P. (2022). The prevalence of comorbid serious mental illnesses and substance use disorders in prison populations: A systematic review and meta-analysis. *Lancet Public Health* 7 e557–e568. 10.1016/S2468-2667(22)00093-7 35660217 PMC9178214

[B7] Barkley R. A. Fischer M. Smallish L. Fletcher K. (2004). Young adult follow-up of hyperactive children: Antisocial activities and drug use. *J. Child Psychol. Psychiatry* 45 195–211. 10.1111/j.1469-7610.2004.00214.x 14982236

[B8] Bebbington P. Jakobowitz S. McKenzie N. Killaspy H. Iveson R. Duffield G. (2017). Assessing needs for psychiatric treatment in prisoners: 1. Prevalence of disorder. *Soc. Psychiatry Psychiatr. Epidemiol.* 52 221–229. 10.1007/s00127-016-1311-7 27878322 PMC5329095

[B9] Borschmann R. Janca E. Carter A. Willoughby M. Hughes N. Snow K. (2020). The health of adolescents in detention: A global scoping review. *Lancet Public Health* 5 e114–e126. 10.1016/S2468-2667(19)30217-8 31954434 PMC7025881

[B10] Bowler N. Phillips C. Rees P. (2018). The association between imported factors and prisoners’ mental health: Implications for adaptation and intervention. *Int. J. Law Psychiatry* 57 61–66. 10.1016/j.ijlp.2018.01.001 29548505

[B11] Bukten A. Lund I. O. Kinner S. A. Rognli E. B. Havnes I. A. Muller A. E. (2020). Factors associated with drug use in prison – results from the Norwegian offender mental health and addition (NorMA) study. *Health Justice* 8:10. 10.1186/s40352-020-00112-8 32399643 PMC7218530

[B12] Bulten E. Nijman H. van der Staak C. (2009). Psychiatric disorders and personality characteristics of prisoners at regular prison wards. *Int. J. Law Psychiatry* 32 115–119. 10.1016/j.ijlp.2009.01.007 19217664

[B13] Cahill B. S. Coolidge F. L. Segal D. L. Klebe K. J. Marle P. D. Overmann K. A. (2012). Prevalence of ADHD and its subtypes in male and female adult prison inmates. *Behav. Sci. Law* 30 154–166. 10.1002/bsl.2004 22496046

[B14] Chang Z. Larsson H. Lichtenstein P. Fazel S. (2015). Psychiatric disorders and violent reoffending: A national cohort study of convicted prisoners in Sweden. *Lancet Psychiatry* 2 891–900. 10.1016/S2215-0366(15)00234-5 26342957 PMC4629414

[B15] Cuetos F. Arribas D. Ramos J. L. (2016). *PROLEC-SE-R. Batería de evaluación de los procesos lectores en secundaria y bachillerato – revisada.* Madrid: TEA Ediciones.

[B16] Cuetos F. Ramos J. L. Ruano E. (2002). *PROESC. Evaluación de los procesos de escritura.* Madrid: TEA Ediciones.

[B17] de Andrade D. Kinner S. A. (2016). Systematic review of health and behavioural outcomes of smoking cessation interventions in prisons. *Tob. Control* 26 495–501. 10.1136/tobaccocontrol-2016-053297 27798322 PMC5574402

[B18] Degenhardt L. Larney S. Kimber J. Gisev N. Farrell M. Dobbins T. (2014). The impact of opioid substitution therapy on mortality post-release from prison: Retrospective data linkage study. *Addiction* 109 1306–1317. 10.1111/add.12536 24612249

[B19] Eggers M. Muñoz J. P. Sciulli J. Crist P. A. (2006). The community reintegration project: Occupational therapy at work in a county jail. *Occup. Ther. Health Care* 20 17–37. 10.1080/J003v20n01_02 23926890

[B20] Einat T. Einat A. (2008). Learning disabilities and delinquency: A study of Israeli prison inmates. *Int. J. Offender Ther. Comp. Criminol.* 52 416–434. 10.1177/0306624X07307352 17909247

[B21] Elbeheri G. Everatt J. Al Malki M. (2009). The incidence of dyslexia among young offenders in Kuwait. *Dyslexia* 15 86–104. 10.1002/dys.361 18433005

[B22] Fayyad J. De Graaf R. Kessler R. Alonso J. Angermeyer M. Demyttenaere K. (2007). Cross-national prevalence and correlates of adult attention-deficit hyperactivity disorder. *Br. J. Psychiatry* 190 402–409. 10.1192/bjp.bp.106.034389 17470954

[B23] Fazel S. Danesh J. (2002). Serious mental disorder in 23000 prisoners: A systematic review of 62 surveys. *Lancet* 359 545–550. 10.1016/S0140-6736(02)07740-1 11867106

[B24] Fazel S. Seewald K. (2012). Severe mental illness in 33,588 prisoners worldwide: Systematic review and meta-regression analysis. *Br. J. Psychiatry* 200 364–373. 10.1192/bjp.bp.111.096370 22550330

[B25] Fernández-Pacheco Alises G. Torres-Jiménez M. Martins P. C. Mendes S. M. V. (2022). Analysing the relationship between immigrant status and the severity of offending behaviour in terms of individual and contextual factors. *Front. Psychol.* 13:915233. 10.3389/fpsyg.2022.915233 35783765 PMC9240772

[B26] First M. B. Gibbon M. Spitzer R. L. Williams J. B. W. Benjamin L. S. (1997a). *Entrevista clínica estructurada para los trastornos de personalidad del eje II del DSM-IV.* Barcelona: Masson.

[B27] First M. B. Spitzer R. L. Williams J. B. W. Gibbon M. (1997b). *Entrevista clínica estructurada para los trastornos del eje I del DSM-IV. Versión clínica (SCID-I-VC).* Barcelona: Masson.

[B28] Gaïffas A. Galéra C. Mandon V. Bouvard M. P. (2014). Attention-deficit/hyperactivity disorder in young French male prisoners. *J. Forensic Sci.* 59 1016–1019. 10.1111/1556-4029.12444 24673682

[B29] Garofalo C. Velotti P. Crocamo C. Carrà G. (2018). Single and multiple clinical syndromes in incarcerated offenders: Associations with dissociative experiences and emotionality. *Int. J. Offender Ther. Comp. Criminol.* 62 1300–1316. 10.1177/0306624X16682325 27913716 PMC5858637

[B30] Haavik J. Halmøy A. Lundervold A. J. Fasmer O. B. (2010). Clinical assessment and diagnosis of adults with attention-deficit/hyperactivity disorder. *Expert Rev. Neurother.* 10 1569–1580. 10.1586/ern.10.149 20925472

[B31] Hamzeloo M. Mashhadi A. Salehi Fadardi J. (2016). The prevalence of ADHD and comorbid disorders in Iranian adult male prison inmates. *J. Atten. Disord.* 20 590–598. 10.1177/1087054712457991 22997358

[B32] Harris P. J. Baltodano H. M. Artiles A. J. Rutherford R. B. (2006). Integration of culture in reading studies for youth in corrections: A literature review. *Educ. Treat. Child* 29 749–778.

[B33] Heller P. Morosan L. Badoud D. Laubscher M. Jimenez Olariaga L. Debbané M. (2022). Prevalence rates and evolution of psychiatric disorders among incarcerated youths in comparison with non-incarcerated youths. *Front. Psychiatry* 12:784954. 10.3389/fpsyt.2021.784954 35069287 PMC8782264

[B34] Hofvander B. Anckarsäter H. Wallinius M. Billstedt E. (2017). Mental health among young adults in prison: The importance of childhood-onset conduct disorder. *BJPsych Open* 3 78–84. 10.1192/bjpo.bp.116.003889 28357134 PMC5362727

[B35] Hughes N. Chitsabesan P. Bryan K. Borschmann R. Swain N. Lennox C. (2017). Language impairment and comorbid vulnerabilities among young people in custody. *J. Child. Psychol. Psychiatry* 58 1106–1113. 10.1111/jcpp.12791 28833100

[B36] Hughes N. Williams P. Chitsabesan P. Davies R. Mounce L. (2012). *Nobody made the connection; the prevalence of neurodisability in young people who offend.* London: Office for the Children’s Commissioner.

[B37] Hughes T. Wilson D. J. (2002). *Reentry trends in the United States.* Washington, DC: Bureau of Justice Statistics.

[B38] Karlsson A. Håkansson A. (2022). Crime-specific recidivism in criminal justice clients with substance use – a cohort study. *Int. J. Environ. Res. Public Health* 19:7623. 10.3390/ijerph19137623 35805282 PMC9265645

[B39] Kessler R. C. Adler L. Barkley R. Biederman J. Conners C. K. Demler O. (2006). The prevalence and correlates of adult ADHD in the United States: Results from the national comorbidity survey replication. *Am. J. Psychiatry* 163 716–723. 10.1176/ajp.2006.163.4.716 16585449 PMC2859678

[B40] Kim J. I. Kim B. Kim B. N. Hong S. B. Lee D. W. Chung J. Y. (2017). Prevalence of psychiatric disorders, comorbidity patterns, and repeat offending among male juvenile detainees in South Korea: A cross-sectional study. *Child Adolesc. Psychiatry Ment. Health* 11:6. 10.1186/s13034-017-0143-x 28115987 PMC5241965

[B41] Knäuper B. Cannell C. F. Schwarz N. Bruce M. L. Kessler R. C. (1999). Improving accuracy of major depression age-of-onset reports in the US national comorbidity survey. *Int. J. Methods Psychiatr. Res.* 8 39–48. 10.1002/mpr.55

[B42] Kooij J. J. S. (2012). *Adult ADHD: Diagnostic assessment and treatment*, 3rd Edn. London: Springer-Verlag. 10.1007/978-1-4471-4138-9

[B43] Krona H. Nyman M. Andreasson H. Vicencio N. Anckarsäter H. Wallinius M. (2017). Mentally disordered offenders in Sweden: Differentiating recidivists from non-recidivists in a 10-year follow-up study. *Nord. J. Psychiatry* 71 102–109. 10.1080/08039488.2016.1236400 27701993

[B44] Lee H. L. Hwang E. J. Wu S. L. Tu W. M. Wang M. H. Chan F. (2018). Employment outcomes after vocational training for people with chronic psychiatric disorders: A multicenter study. *Am. J. Occup. Ther.* 72:7205195010. 10.5014/ajot.2018.028621 30157009

[B45] Lewis D. O. Shanok S. S. Balla D. A. Bard B. (1980). Psychiatric correlates of severe reading disabilities in an incarcerated delinquent population. *J. Am. Acad. Child Psychiatry* 19 611–622. 10.1016/S0002-7138(09)60965-1 7204793

[B46] Lindgren M. Jensen J. Dalteg A. Meurling A. W. Ingvar D. H. Levander S. (2002). Dyslexia and AD/HD among Swedish prison inmates. *J. Scan. Stud. Criminol. Crime Prev.* 3 84–95. 10.1080/140438502762467227

[B47] Macciò A. Meloni F. R. Sisti D. Rocchi M. B. Petretto D. R. Masala C. (2015). Mental disorders in Italian prisoners: Results of the REDiMe study. *Psychiatry Res.* 225 522–530. 10.1016/j.psychres.2014.11.053 25534756

[B48] Martin M. S. Colman I. Simpson A. I. McKenzie K. (2013). Mental health screening tools in correctional institutions: A systematic review. *BMC Psychiatry* 13:275. 10.1186/1471-244X-13-275 24168162 PMC4231452

[B49] Mohr-Jensen C. Steinhausen H. C. (2016). A meta-analysis and systematic review of the risks associated with childhood attention-deficit hyperactivity disorder on long-term outcome of arrests, convictions, and incarcerations. *Clin. Psychol. Rev.* 48 32–42. 10.1016/j.cpr.2016.05.005 27390061

[B50] Moreno-Küstner B. Martín C. Pastor L. (2018). Prevalence of psychotic disorders and its association with methodological issues. A systematic review and meta-analyses. *PLoS One* 13:e0195687. 10.1371/journal.pone.0195687 29649252 PMC5896987

[B51] Mumola C. J. Karberg J. C. (2006). *Drug use and dependence, state and federal prisoners, 2004*. Bureau of justice statistics special report NCJ 213530. Washington, DC: Office of Justice Programs. 10.1037/e560272006-001

[B52] Mundt A. P. Baranyi G. Gabrysch C. Fazel S. (2018). Substance use during imprisonment in low- and middle-income countries. *Epidemiol. Rev.* 40 70–81. 10.1093/epirev/mxx016 29584860 PMC5982797

[B53] Nacher M. Ayhan G. Arnal R. Basurko C. Huber F. Pastre A. (2018). High prevalence rates for multiple psychiatric conditions among inmates at French Guiana’s correctional facility: Diagnostic and demographic factors associated with violent offending and previous incarceration. *BMC Psychiatry* 18:159. 10.1186/s12888-018-1742-7 29843661 PMC5975419

[B54] Navarro-Mateu F. Tormo M. J. Salmerón D. Vilagut G. Navarro C. Ruíz-Merino G. (2015). Prevalence of mental disorders in the south-east of Spain, one of the European regions most affected by the economic crisis: The cross-sectional PEGASUS-Murcia project. *PLoS One* 10:e0137293. 10.1371/journal.pone.0137293 26394150 PMC4578930

[B55] Peled-Laskov R. Shoham E. Cojocaru L. (2019). Work-related intervention programs: Desistance from criminality and occupational integration among released prisoners on parole. *Int. J. Offender Ther. Comp. Criminol.* 63 2264–2290. 10.1177/0306624X19845762 31064230

[B56] Petersilia J. (2005). “From cell to society: Who is returning home?,” in *Prison reentry and crime in America*, eds Travis J. Visher C. (New York, NY: Cambridge University Press), 15–49. 10.1017/CBO9780511813580.002

[B57] Philipp-Wiegmann F. Rösler M. Clasen O. Zinnow T. Retz-Junginger P. Retz W. (2018). ADHD modulates the course of delinquency: A 15-year follow-up study of young incarcerated man. *Eur. Arch. Psychiatry Clin. Neurosci.* 268 391–399. 10.1007/s00406-017-0816-8 28612143

[B58] Puljević C. Kinner S. A. de Andrade D. (2017). Extending smoking abstinence after release from smoke-free prisons: Protocol for a randomised controlled trial. *Health Justice* 5:1. 10.1186/s40352-016-0046-6 28116579 PMC5256626

[B59] Raj A. T. Patil S. Sarode S. Salameh Z. (2018). P-hacking: A wake-up call for the scientific community. *Sci. Eng. Ethics* 24 1813–1814. 10.1007/s11948-017-9984-1 29071570

[B60] Ramos-Quiroga J. A. Nasillo V. Richarte V. Corrales M. Palma F. Ibáñez P. (2019). Criteria and concurrent validity of DIVA 2.0: A semi-structured diagnostic interview for adult ADHD. *J. Atten. Disord.* 23 1126–1135. 10.1177/1087054716646451 27125994

[B61] Reid G. Kirk J. (2001). *Dyslexia in adults: Education and employment.* Chichester: Wiley.

[B62] Román-Ithier J. C. González R. A. Vélez-Pastrana M. C. González-Tejera G. M. Albizu-García C. E. (2017). Attention deficit hyperactivity disorder symptoms, type of offending and recidivism in a prison population: The role of substance dependence. *Crim. Behav. Mental Health* 27 443–456. 10.1002/cbm.2009 27455899 PMC5269538

[B63] Satterfield J. H. Faller K. J. Crinella F. M. Schell A. M. Swanson J. M. Homer L. D. (2007). A 30-year prospective follow-up study of hyperactive boys with conduct problems: Adult criminality. *J. Am. Acad. Child Adolesc. Psychiatry* 46 601–610. 10.1097/chi.0b013e318033ff59 17450051

[B64] Scott D. A. Gignac M. Kronfli R. N. Ocana A. Lorberg G. W. (2016). Expert opinion and recommendations for the management of attention-deficit/hyperactivity disorder in correctional facilities. *J. Correct. Health Care* 22 46–61. 10.1177/1078345815618392 26672119

[B65] Segal D. L. Mueller A. Coolidge F. L. (2012). “Structured and semistructured interviews for differential diagnosis: Fundamentals, applications, and essential features,” in *Adult psychopathology and diagnosis*, eds Hersen M. Beidel D. C. (New York, NY: Wiley), 91–115.

[B66] Simon V. Czobor P. Bálint S. Mészáros A. Bitter I. (2009). Prevalence and correlates of adult attention-deficit hyperactivity disorder: Meta-analysis. *Br. J. Psychiatry* 194 204–211. 10.1192/bjp.bp.107.048827 19252145

[B67] Simonoff E. Elander J. Holmshaw J. Pickles A. Murray R. Rutter M. (2004). Predictors of antisocial personality. Continuities from childhood to adult life. *Br. J. Psychiatry* 184 118–127. 10.1192/bjp.184.2.118 14754823

[B68] Slade K. Forrester A. (2013). Measuring IPDE-SQ personality disorder prevalence in pre-sentence and early-stage prison populations, with sub-type estimates. *Int. J. Law Psychiatry* 36 207–212. 10.1016/j.ijlp.2013.04.018 23627987

[B69] Solberg B. S. Halmøy A. Engeland A. Igland J. Haavik J. Klungsøyr K. (2018). Gender differences in psychiatric comorbidity: A population-based study of 40 000 adults with attention deficit hyperactivity disorder. *Acta Psychiatr. Scand.* 137 176–186. 10.1111/acps.12845 29266167 PMC5838558

[B70] Steadman H. J. Mulvey E. P. Monahan J. Robbins P. C. Appelbaum P. S. Grisso T. (1998). Violence by people discharged from acute psychiatric inpatient facilities and by others in the same neighborhoods. *Arch. Gen. Psychiatry* 55 393–401. 10.1001/archpsyc.55.5.393 9596041

[B71] Streb J. Lutz M. Dudeck M. Klein V. Maaß C. Fritz M. (2022). Are women really different? Comparison of men and women in a sample of forensic psychiatric inpatients. *Front. Psychiatry* 13:857468. 10.3389/fpsyt.2022.857468 35401259 PMC8985759

[B72] Talari K. Goyal M. (2020). Retrospective studies – utility and caveats. *J. R. Coll. Physicians Edinb.* 50 398–402. 10.4997/jrcpe.2020.409 33469615

[B73] van Buitenen N. van den Berg C. J. W. Meijers J. Harte J. M. (2020). The prevalence of mental disorders and patterns of comorbidity within a large sample of mentally ill prisoners: A network analysis. *Eur. Psychiatry* 63:e63. 10.1192/j.eurpsy.2020.63 32522312 PMC7355171

[B74] Vélez-Pastrana M. C. González R. A. Ramos-Fernández A. Ramírez Padilla R. R. Levin F. R. Albizu García C. (2020). Attention deficit hyperactivity disorder in prisoners: Increased substance use disorder severity and psychiatric comorbidity. *Eur. Addict. Res.* 26 179–190. 10.1159/000508829 32615575

[B75] Vicens E. Tort V. Dueñas R. M. Muro A. Pérez-Arnau F. Arroyo J. M. (2011). The prevalence of mental disorders in Spanish prisons. *Crim. Behav. Ment. Health* 21 321–332. 10.1002/cbm.815 21706528

[B76] Vogel S. A. (1997). *College students with learning disabilities: A handbook.* Pittsburgh, PA: Learning Disabilities Association of America.

[B77] White L. M. Lau K. S. Aalsma M. C. (2016). Detained adolescents: Mental health needs, treatment use, and recidivism. *J. Am. Acad. Psychiatry Law* 44 200–212. 27236176

[B78] Young S. Cocallis M. (2019). Attention deficit hyperactivity disorder (ADHD) in the prison system. *Curr. Psychiatry Rep.* 21:41. 10.1007/s11920-019-1022-3 31037396

[B79] Young S. Moss D. Sedgwick O. Fridman M. Hodgkins P. (2015). A meta-analysis of the prevalence of attention deficit hyperactivity disorder in incarcerated population. *Psychol. Med.* 45 247–258. 10.1017/S0033291714000762 25066071 PMC4301200

[B80] Young S. Wells J. Gudjonsson G. H. (2011). Predictors of offending among prisoners: The role of attention-deficit hyperactivity disorder and substance use. *J. Psychopharmacol.* 25 1524–1532. 10.1177/0269881110370502 20558498

[B81] Yu R. Geddes J. R. Fazel S. (2012). Personality disorders, violence, and antisocial behavior: A systematic review and meta-regression analysis. *J. Pers. Disord.* 26 775–792. 10.1521/pedi.2012.26.5.775 23013345

[B82] Zhao Y. Messner S. F. Liu J. Jin C. (2019). Prisons as schools: Inmates’ participation in vocational and academic programs in Chinese prisons. *Int. J. Offender Ther. Comp. Criminol.* 63 2713–2740. 10.1177/0306624X19861051 31288609

